# Economic Game Theory to Model the Attenuation of Virulence of an Obligate Intracellular Bacterium

**DOI:** 10.3389/fcimb.2016.00086

**Published:** 2016-08-25

**Authors:** Damian Tago, Damien F. Meyer

**Affiliations:** ^1^La Recherche Agronomique Pour le Développement (CIRAD), UMR Contrôle des Maladies Animales, Exotiques et Émergentes (CMAEE)Petit-Bourg, France; ^2^Institut National de la Recherche Agronomique, UMR1309 CMAEEMontpellier, France

**Keywords:** obligate intracellular bacteria, *Ehrlichia ruminantium*, co-evolution, game theory, virulence attenuation

## Abstract

Diseases induced by obligate intracellular pathogens have a large burden on global human and animal health. Understanding the factors involved in the virulence and fitness of these pathogens contributes to the development of control strategies against these diseases. Based on biological observations, a theoretical model using game theory is proposed to explain how obligate intracellular bacteria interact with their host. The equilibrium in such a game shows that the virulence and fitness of the bacterium is host-triggered and by changing the host's defense system to which the bacterium is confronted, an evolutionary process leads to an attenuated strain. Although, the attenuation procedure has already been conducted in practice in order to develop an attenuated vaccine (e.g., with *Ehrlichia ruminantium*), there was a lack of understanding of the theoretical basis behind this process. Our work provides a model to better comprehend the existence of different phenotypes and some underlying evolutionary mechanisms for the virulence of obligate intracellular bacteria.

## Background

Obligate intracellular bacteria are characterized by reproducing only within the cells of other organisms and are cause of several diseases that affect different type of hosts, from humans to protists. Obligate intracellular bacteria include, amongst others, *Chlamydia*, rickettsial pathogens (i.e., *Rickettsia, Anaplasma, Ehrlichia*), and *Coxiella*; and the burden of the diseases they induce can be very large. For instance, global *Chlamydia* infections lead to 714,000 disability-adjusted life years (DALYs) lost in 2010 (Murray et al., 2013), and in 1993 the USDA estimated that the cost of a Heartwater outbreak (disease caused by *Ehrlichia ruminantium*) in the USA could exceed US$ 760 million in losses annually (Gersabeck, 1994).

A pathogen such as *Ehrlichia*, uses tightly regulated pathogenicity determinants to hijack host cellular processes and replicate inside the cell (extensively reviewed in Moumène and Meyer, 2016). Thus, the invasion of the cell requires bacterial adhesion to a specific receptor. Immediately after endocytosis, *Ehrlichia* establishes a safe replicating niche by evading host innate immunity through the induction of dedicated secretion systems and secretion of corresponding type I and IV effector proteins. Bacterial replication itself needs nutrients from the host and occurs thanks to the subversion of host cell machineries (e.g., manipulation of autophagy or cholesterol pathways; Barnewall et al., 1997; Niu et al., 2008; Xiong et al., 2008; Wakeel et al., 2009; Liu et al., 2012; Xiong and Rikihisa, 2012). Recent transcriptomic studies on *E. ruminantium* infectious form provide evidence of the expression of genes involved in the bacterial defense and invasion systems, showing that *Ehrlichia* allocates a significant part of its energetic resources to virulent purposes (Pruneau et al., 2012).

During *Ehrlichia* infection, the host's extracellular defense system involves cellular immunity with macrophages, CD4^+^ T-cells and production of IL-17 by peripheral blood leukocytes (Totté et al., 1999; Bitsaktsis et al., 2004; McGill et al., 2016). Dendritic cells also play a role in immunity against *Ehrlichia* (Nandi et al., 2009). Despite the obligate intracellular nature of *Ehrlichia*, the role of humoral immunity has also been described using tandem repeat proteins or outer membrane proteins as antigens (Yager et al., 2005; reviewed in Rikihisa, 2015).

On the other hand, the intracellular defense system is characterized by the induction of the phagolysosomal fusion, the generation of reactive oxygen species, the activation of proinflammatory chemokines/cytokines, IFN-gamma activation and signaling, the production of antimicrobial peptides and the induction of host cell apoptosis (Rikihisa, 2010; de la Fuente et al., 2016). Therefore, the bacterial success to replicate should depend on whether it faces both the extra- and intra-cellular host's defense systems or just one of them.

It has been shown that *in vitro* passaging of the obligate intracellular bacteria *E. ruminantium* can lead to an attenuated strain (Jongejan, 1991; Zweygarth et al., 2005; Pilet et al., 2012; Marcelino et al., 2015). *In vitro* and *in vivo* infection essays have shown that the virulent strain is highly pathogenic to goats and toxic to host cells *in vitro* just after infection, while the attenuated strain is not lethal in goats and has a shorter life cycle (Pilet et al., 2012; Marcelino et al., 2015). Although, this mechanism has been essential for the development of inactivated vaccines against obligate intracellular bacterial infections, such as Heartwater (Zweygarth et al., 2005, 2008; Vachiéry et al., 2006), there is little understanding of the attenuation process and no formal theory has been proposed.

Moreover, proteomic studies have increased the understanding of the main differences that exist between the attenuated and virulent strains of *E. ruminantium* (Marcelino et al., 2015). While 292 proteins (80% of the proteome) were found to be common between the attenuated and the virulent strains, there is a significant number of strain-specific proteins expressed (49 proteins for the virulent and 72 for the attenuated). A higher number of proteins useful to fuel specific processes involved in virulence were detected in the virulent strain, while a higher number of proteins associated with metabolism of lipids and amino acids, protein processing, and biosynthesis of co-factors could relate to higher growth rate of the attenuated strain *in vitro* (Marcelino et al., 2015). This suggests that part of the energy available to the virulent strain is used to evade the host's defense system, while the attenuated strain seems to reserve this energy to replication purposes. In other bacteria, some molecular determinants have been associated with the attenuation mechanism. For instance, in the case of *Rickettsia prowazekii*, mutations of a single gene (*smt*) that encodes a SAM-methyltransferase led to the attenuation of the bacteria (Liu et al., 2014). When comparing different phenotypes of *R. prowazekii*, transcriptional and proteomic analyses have shown that surface protein expression and protein methylation varied with virulence (Bechah et al., 2010).

## Game theory

Game theory has gained popularity among biologists to analyze biological interactions involving two or more organisms and to elucidate evolutionary consequences of interactions (Smith, 1979), and has been proposed as a more appropriate approach to analyze evolutionary puzzles than optimization algorithms (Nowak and Sigmund, 2004). In evolutionary game theory, the success of a strategy is not just determined by how good the strategy is, but the presence of other alternative strategies and the frequency at which they are employed within a competing population play a very important role in determining which population will eventually become dominant. An evolutionary stable strategy leads to equilibrium such that once the strategy is fixed in a population, natural selection is sufficient to avoid other strategies to invade and change the equilibrium (Maynard-Smith, 1983).

This approach has been used to model complex situations for which natural environments, spatial and temporal variability in resource abundance and quality can influence biotic interactions (Nowak and May, 1992), such as competition for territories between organisms (Hammerstein, 1981), the location of pathogenic bacteria during persistent infection (Eswarappa, 2009), or the status of different symbiotic relationships (Renaud and Meeüs, 1991).

In the context of obligate intracellular bacteria, game theory can help to understand how the host-pathogen interaction leads to an equilibrium that determines the characteristics of the bacteria. Understanding the host-pathogen interaction with obligate intracellular bacteria would assist in the identification of evolutionary differences between strains of the same bacteria and elucidate mechanisms that can lead to the control of such organisms. Experimental data regarding the genes or proteins involved in bacterial pathogenesis is crucial to depict the strategy implemented by a bacterium when infecting its host.

## The evasion-replication game for *E. ruminantium*

We propose a repeated host-pathogen game to analyze the evolution of obligate intracellular pathogenic bacteria in terms of virulence and fitness. The results obtained from solving the game enable us to propose one scenario for understanding why *in vitro* passaging leads to the attenuation of the bacterium, and provides some insights on the phenotypic differences observed between strains of *E. ruminantium*.

There are two players in this game: the intracellular bacterium and the host. Each player chooses a strategy and the payoff of each player not only depends on the strategy that the player chose, but also on the strategy that his opponent selected. The strategies are the following:

- Intracellular bacterium:
  ◦ Strategy B1: Evasion of host's defense before replication—Before trying to replicate, the bacterium pays a cost *c* in terms of energy to evade the host's extracellular defense system.
  ◦ Strategy B2: Replication without evasion of extracellular immune response—concentrates all the energy to replicate, without spending energy in evading host's defense system.
- Host:
  ◦ Strategy H1: Incomplete defense—Only intracellular defense mechanisms are expressed.
  ◦ Strategy H2: Complete defense—Both, intra and extracellular defense mechanisms are expressed.

It is to note that a situation characterized by a host adopting strategy H1 (incomplete defense) represents the *in vitro* scenario; while a host adopting strategy H2 (complete defense) represents the *in vivo* scenario.

### Assumptions

A bacterium interacting with a host that follows the incomplete defense strategy H1 succeeds in replicating with probability equal to 1 by adopting strategy B1 (evasion before replication) and with probability equal to *r* by adopting strategy B2 (replication without evasion). On the other hand, a bacterium interacting with a host following the complete defense strategy H2 can succeed in replicating only after evading the host's defense system, with a probability of success equal to *s*. With *L* being the gain of the bacterium if it succeeds to replicate, assume that $r>\frac{L-c}{L}$, i.e., whenever the bacterium faces the incomplete defense system, its optimal response is replicating without evasion (B2).

### Payoffs

- Bacterium:
  ◦ If the host uses the incomplete defense system (strategy H1), let *P*_*b*_ be the payoff of the bacteria, *L* the gain of the bacterium if it succeeds to replicate, and *c* the energy cost of evading the host's defense system, then the bacterium's expected payoffs are:
    ▪ Evasion before replication strategy (strategy B1): *P*_*b*_ = *L* − *c*
    ▪ Replication without evasion strategy (strategy B2): *P*_*b*_ = *rL*
  ◦ If the host uses the complete defense system:
    ▪ Evasion before replication strategy (strategy B1): *P*_*b*_ = *sL* − *c*
    ▪ Replication without evasion strategy (strategy B2): *P*_*b*_ = 0
- Host:

The payoff of the host is a loss function proportional to the gain of the bacterium, i.e., *P*_*H*_ = − *kP*_*b*_, with *k* > 0.

Therefore, the normal form game is:

**Table:** 

	**Incomplete defense (intracellular)**	**Complete defense (intra + extracellular)**
Evasion before replication	L − c, −k(L − c)	sL − c, −k(sL − c)
Replication without evasion	rL, −krL	0, 0

## Game's equilibrium and discussion

**Figure 1:**
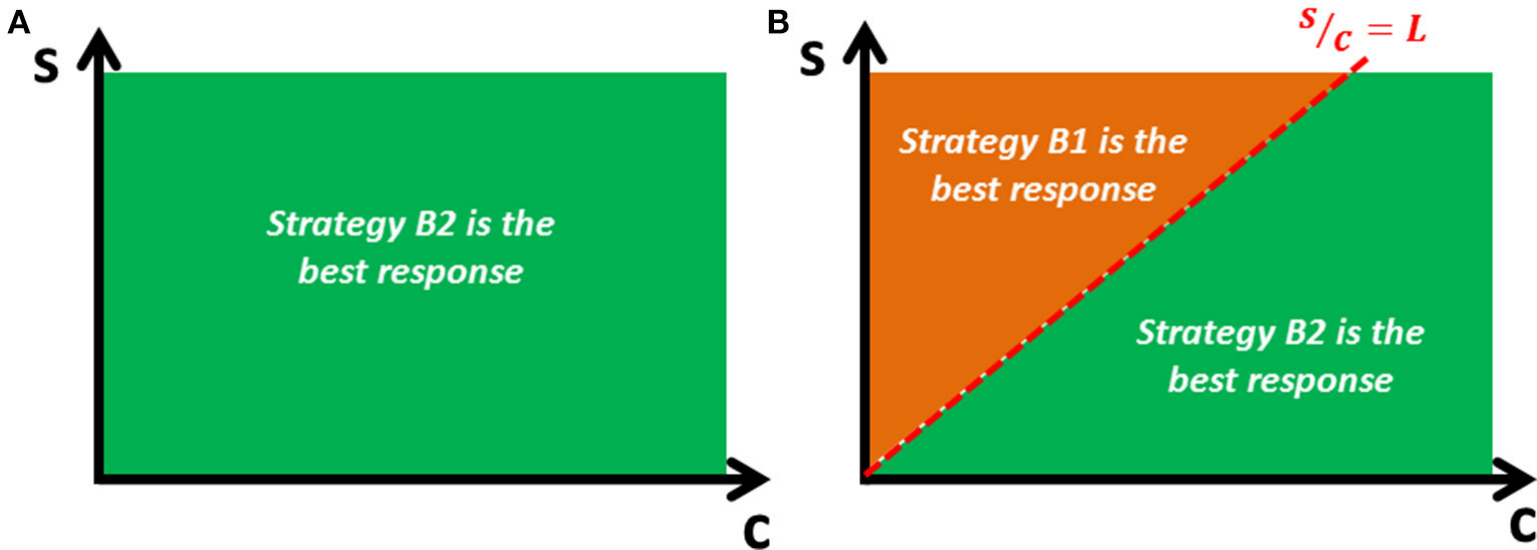
**Bacterium's best response**. The optimal strategy for the bacterial player depends on the cost-efficiency parameters (s and c). **(A)** If the bacterium faces the host's incomplete defense system (only intracellular), the best response is to replicate without evading (strategy B2). **(B)** If the bacterium faces the host's complete defense system (both, intra- and extracellular), the best response is to evade before replicating (strategy B1) if the efficiency parameter [s] is sufficiently high relative to the cost parameter [c].

For the host, activating the complete defense system (intra- and extra-cellular) is a dominant strategy, whereas the bacterium has no dominant strategy. Thus, when facing a host that adopts the incomplete defense system (strategy H1), the best response for the bacterium is to replicate without evading (since $r\;>\;\frac{L-c}{L}$), but when facing the host's complete defense system (strategy H2), the best response is the evasion before replication strategy if the probability of success is sufficiently high ($s\;\geq \;\frac{c}{L}$) (Figure 1). Therefore, there is a unique pure strategy Nash equilibrium: (Evasion before replication, Complete defense). Let's call this equilibrium the “virulent equilibrium,” which is the outcome observed in nature and is characterized by a high level of virulence that can lead to the death of the animal.

It has been shown that *E. ruminantium* becomes attenuated after several passages in laboratory conditions. In our game, “*in vitro*” conditions were captured by restricting the host player exclusively to his incomplete defense system (intracellular). In this case, the bacterium has no interest in paying the cost of the virulent strategy, since it can replicate without the need to evade the host's defense system. Thus, the “attenuated equilibrium” is: (Replication without evasion, Incomplete defense). Note that this equilibrium is not observed in nature and it can only be achieved in a scenario in which the host does not have access to the complete defense system (such as the *in vitro* conditions). At this equilibrium, the speed at which the bacterium replicates increases and overcomes the baseline replication speed observed at the virulent equilibrium.

According to our game, virulence is not just a reflect of the bacterium's characteristics but it is the outcome of the interaction between the bacterium and the host defense system. When the bacterium faces the host's complete defense system, the best strategy is to evade the defense system before replicating. This increases the probability of successful replication due to the delivery of effectors that counteract the host immune response and scavenge the host cell. Thus, bacterial populations that invest energy in evading the host's defense system present a replicative advantage *in vivo* and end up being dominant over less virulent bacterial populations. However, this strategy requires the consumption of energy that decreases the final payoff of the bacterium.

In a repeated game framework, we can imagine that the cost of the evading strategy (ability to evade the host's immune responses) depends on how frequently this strategy is chosen (“if you fail to practice your art, it will soon disappear”). If the cost *c* is an increasing function of the number of times that the replication-without-evasion strategy (strategy B2) has been chosen in the past [i.e., *c* = *c*(*n*) with *c*′(*n*) > 0, where *n* is the number of times the replication-without-evasion strategy has been chosen], the fact that *Ehrlichia* becomes attenuated after several passages and unable to survive *in vivo* afterwards can be explained: when facing the *in vitro* conditions, the best strategy of *Ehrlichia* is replicating without evasion (strategy B2). If the bacterium keeps facing the *in vitro* conditions for a while, the cost of the evading strategy (strategy B1) increases, and the cost can become sufficiently high (*c* > *sL*) such that the replication-without-evasion strategy becomes dominant (for the complete game). Therefore, even if those bacteria face again the complete game (i.e., the host activating both defense systems), the dominant strategy will not change, the bacterium will not be able to survive the host's defense system, and the host will survive.

In evolutionary terms, bacterial subpopulations genetically predisposed to invest fewer resources in evading their host's defense system are more efficient in replicating when facing the *in vitro* environment. By passaging the bacteria *in vitro*, the selection pressure picks for the fittest bacteria, which in this case are those overexpressing genes/proteins associated with replication and not evasion of the host's extracellular immune defense.

**Figure 2:**
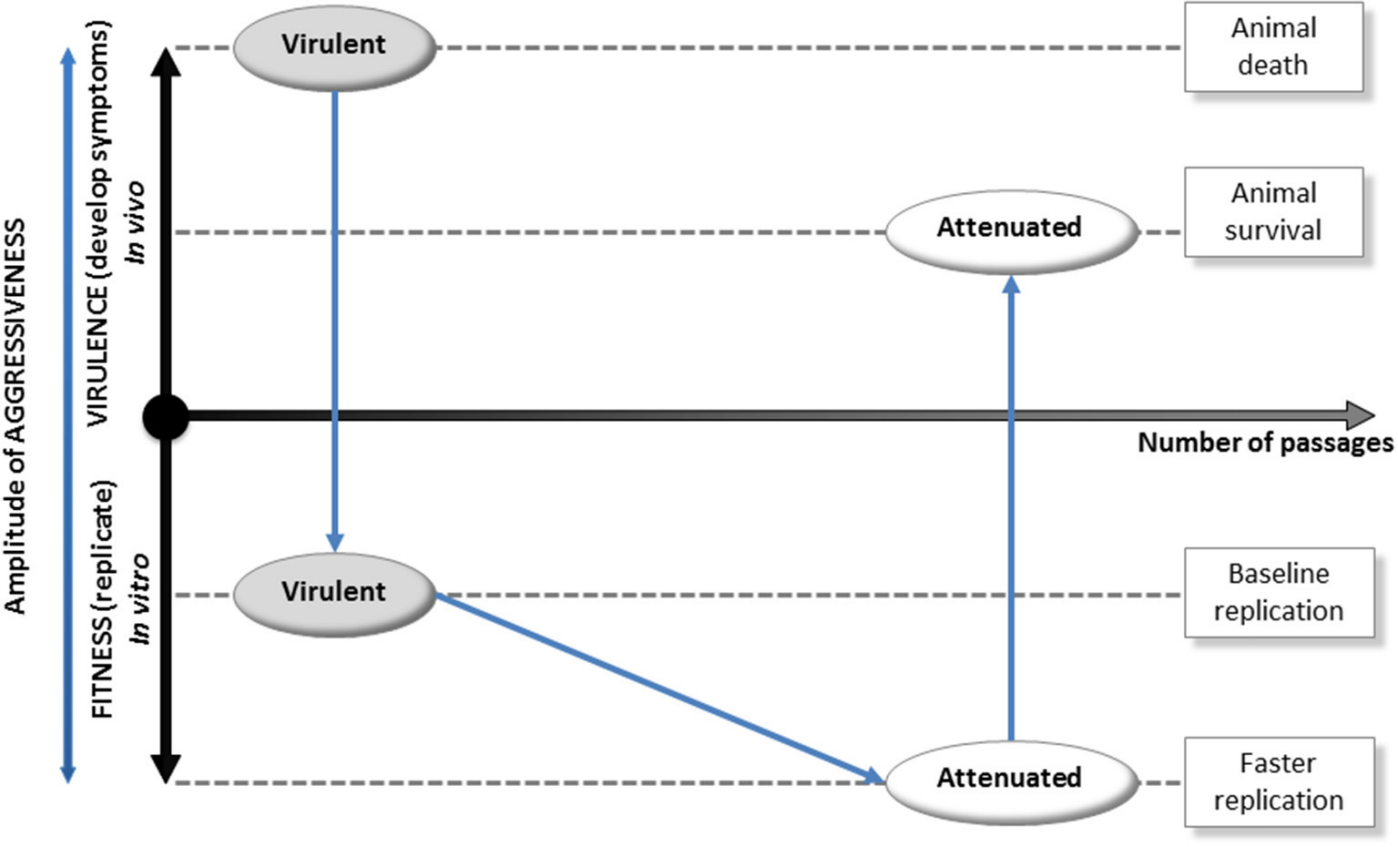
**Evolution of host-triggered aggressiveness in an obligate intracellular pathogenic bacterium**. In this scheme, the ultimate amplitude of aggressiveness is proportional to virulence (ability to induce symptoms and evading the host's immune system) and fitness (ability to replicate inside the cell *in vitro*). First, a virulent strain that sickens the animal delivers effector proteins (i) to counteract host immune response and (ii) to scavenge the host cell. Effector synthesis is an energy-consuming process that leads to a moderate fitness *in vitro*(development cycle of 5 days for virulent Gardel strain). Second, by the mean of iterative passages *in vitro*, the resulting attenuated strain gained skills to grow in cell culture but lost the ability to hijack host immunity and is eliminated when injected in the animal. Thus, this strain exhibits a greater fitness *in vitro* (development cycle of 4 days for attenuated Gardel strain) but a decreased virulence once inside the animal (host's success in eliminating the bacterium). This behavior highlights the co-evolutionary arms race between a bacterial pathogen and its host.

We propose a model where the evolution of *E. ruminantium*'s aggressiveness is triggered by the host. The amplitude of the aggressiveness of the bacterium is proportional to its virulence (ability to induce symptoms) and its fitness (ability to replicate inside the cell *in vitro*; Figure 2). A virulent strain counteracts the host immune response, scavenges the host cell, and ends up leading to the death of the animal. This strategy is associated with the production of proteins that consumes energy and leads to a moderate fitness (development cycle of 5 days for the virulent Gardel strain of *E. ruminantium*; Marcelino et al., 2015). Then, by the mean of iterative *in vitro* passages, the resulting strain increases its speed of replication but ends up losing the ability to hijack host immunity. This attenuated strain exhibits a greater fitness *in vitro* (development cycle of 4 days for attenuated Gardel strain, Marcelino et al., 2015) but a diminished virulence once inside the animal (animal survival), leading to its elimination when confronted to the animal's immune system. This behavior illustrates the molecular co-evolutionary arms race between a pathogen and its host.

In the laboratory it has been shown that the Gardel strain is attenuated after 237 passages, while the Senegal strain is already attenuated after just 64 passages and the Welgevonden strain needs only 43 passages to attenuate (Zweygarth et al., 2005; Pilet et al., 2012; Marcelino et al., 2015). These differences can be explained by slightly modifying the game: Instead of assuming $r\;>\;\frac{L-c}{L}$, consider *r* a random variable such that at every period $r\;=\;r^{+}>\frac{L-c\left(0\right)}{L}$ with probability *q* and $r\;=\;r^{-}\;<\;\frac{L-c\left(0\right)}{L}$ with probability *1-q*. If the probability *q* is strain-specific such that 0 < *q*_*Gardel*_ < *q*_*Senegal*_ < *q*_*Welgevonden*_ < 1, Senegal's number of periods required to stabilize the attenuated equilibrium is smaller than Gardel's but greater than Welgevonden's.^1^

An alternative explanation would be associated to differences in the strains' efficiency at investing energy resources into mechanisms to evade their host's extracellular defense system. According to our model, strains or subpopulations of bacteria that are very efficient in evading the host defense responses (small *c*) would be hardest to attenuate. In models such as the *Neisseria gonorrhoeae*, regulators of two-component systems have been identified as determinants of virulence (Atack et al., 2013). Strains or mutants lacking this type of master regulators, which are associated with the efficiency in using energy resources, should be easier to attenuate.

**Figure 3:**
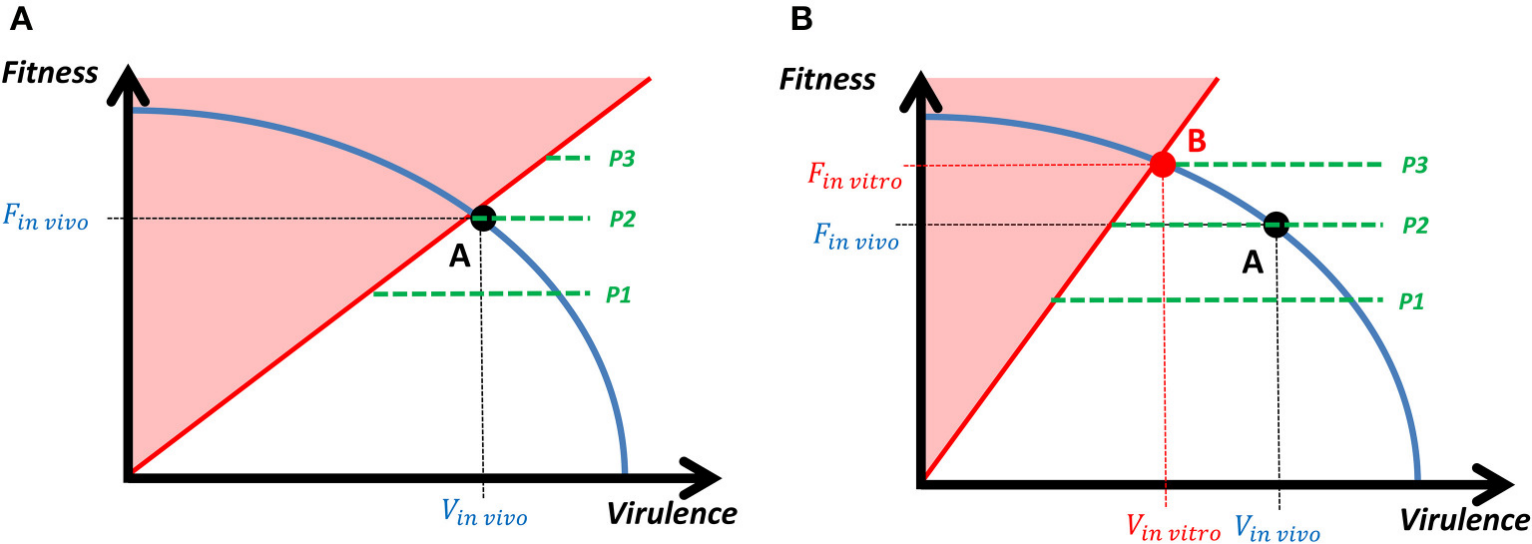
**Transition from ***in vivo*** to ***in vitro*** equilibrium**. The tradeoff between fitness and virulence is represented by the downward sloping efficient frontier (blue line), and the green dotted lines represent the indifference curves. Each indifference curve represents the combination of fitness and virulence that provide the same payoff to the bacteria and we assume that the payoff landscape is independent on the virulence and increasing on fitness, which determines the form of the indifference curves (P1 < P2 < P3). **(A)** When facing the *in vivo* conditions (intra- and extracellular defense systems are adopted by the host) the payoff of the bacterium in the red zones is equal to zero (if the bacteria are too virulent, they will kill the host along with the bacteria themselves; if the bacteria are not virulent enough relative to their fitness, they will be easily detected by the host's immune system and eliminated), and the optimal strategy for the bacterium is characterized by the tangency point between the highest indifference curve and the efficient frontier (point A). **(B)** When moving to the *in vitro* conditions (only intracellular defense system is adopted by the host), the zone for which the bacterium's payoff is reduced, so the equilibrium changes (point B) and the bacterium achieves a higher payoff (P3 > P2). However, when the bacterium faces back the *in vivo* conditions, the equilibrium point B gets into the red zone. This means that the bacteria are not virulent enough (relative to their fitness) to evade the host's immune system and they are eliminated.

The transition from the *in vivo* to the *in vitro* equilibrium can be represented in a continuous framework, in which a bacterium selects the optimal level of fitness and virulence according to its energy constraint (Figure 3). *In vivo*, bacteria with very high fitness but not enough virulence will be easily detected by the complete defense system and eliminated (red zone in Figure 3A). However, the change in the host's strategy (*in vitro* conditions) reduces the area in which the bacterium is eliminated and a new optimal strategy that leads to the attenuated strain is established, which is characterized by a lower virulence and a higher fitness (Figure 3B). The fitness-virulence combination of the attenuated strain belongs to the range of strategies that the host's complete defense system is able to cope with. When the attenuated strain faces again the *in vivo* conditions, its higher fitness leads to a high replication rate that makes easy for the host's immune system to detect the bacterium. Moreover, the lower virulence of the new equilibrium makes the bacterium unable to evade the host's complete defense system, which ends up eliminating the infection.

The virulent strain of *E. ruminantium* expresses a high number of chaperones and proteins to avoid oxidative stress and to overwhelm the humoral immune response (Marcelino et al., 2015). On the contrary, the attenuated strain overexpresses proteins related to rapid bacterial replication (Marcelino et al., 2015). In our model, the payoff of expressing proteins related to virulence decreases when the bacterium does not face the host's extracellular immune response. This leads to a change in the bacterium's optimal strategy, in which the payback of allocating resources for the expression of proteins involved in bacterial replication is higher than the return obtained from the expression of proteins involved in host manipulation and virulence in general.

The change of equilibrium is explained by the competition and selection of the fittest population. When the bacterium is facing the host's incomplete defense system (strategy H1), the subpopulation characterized by trying to replicate without evading (strategy B2) gains a comparative advantage relative to the subpopulation characterized by evading the host immune system before trying to replicate (strategy B1). This advantage, coming from the energy saved associated to strategy B2, makes the population evolve to an equilibrium dominated by bacteria that follow strategy B2, i.e., the attenuated bacteria that reach the maximum fitness to reproduce *in vitro*. Once this population is faced back against the host's complete defense system (strategy H2), the diminishing virulence obtained during the evolutionary process makes the bacterium unable to go back to the original equilibrium (the bacterium has no time to set up strategy B1 before being eliminated by the host, as observational data has shown).

The results of our model suggest that at least part of the difference in virulence levels among strains can be attributed to the co-evolution between host and pathogen. Thus, bacteria facing tolerant or resistant hosts, with sophisticated and effective immune systems, need to allocate a good amount of energetic resources in evading their host's defenses, leading them to high virulence levels. When naïve or susceptible hosts are challenged with these bacteria, the expected outcome would be high levels of morbidity/mortality. However, if the interaction between the pathogen and the susceptible hosts is for long-term, the less virulent bacteria subpopulations that use more resources for replication (and less for evading the host's immune system) should have an evolutionary advantage and the general levels of virulence in the bacterial population are expected to decrease.

A similar reasoning applies to experiments showing how bacteria adapt to the interaction with immunocompromised hosts by reducing their virulence and increasing bacterial within-host fitness (Jansen et al., 2015). The bacteria can make a transition from pathogenicity to commensalism by selecting the avirulent strategy, which allows the bacteria to maximize the use of host resources.

Game theory has been used to analyse problems in cell and molecular biology with a broad range of objectives such as understanding metabolic processes, including the production of costly public goods by cells or cross-feeding, or analysing different competition and interaction models between cellular populations (Hummert et al., 2014).

On the host-pathogen interaction, the concept of Evolutionary Stable Strategies (ESS) has been widely used to characterize the host-pathogen interaction at equilibrium. This approach simplifies the solution of mathematical models developed to analyse the trade-off hypothesis of virulence taking into account immunopathology and how pathogens' virulence evolves (Day et al., 2007). The ESS approach was also used to conciliate the theory with the empirical work about the variance observed in tolerance within hosts by introducing a cost on host fitness (fertility) associated with investing in defense (Best et al., 2008).

A comparable model to ours have been used to characterize two host-pathogen relationships (the killer relationship and diplomat relationship) as potential equilibria that lead to different ways of evolution (coevolution vs. concomitant evolution), and to understand the relevant parameters for reaching a specific equilibria (Renaud and Meeüs, 1991).

Our contribution in the work presented herein relies on the characterization of the transition from a virulent to an attenuated equilibrium (attenuation process). Moreover, our study investigates the evolution of an obligate intracellular bacterium by decoupling the intracellular vs. extracellular selective pressure. Thus, our results highlight the important role that the host plays in the virulence attenuation process of obligate intracellular bacteria.

The proposed game allows us to understand the evolutionary process that leads to the observed virulence/fitness levels of attenuated or virulent obligate intracellular bacteria, such as *E. ruminantium*, in *in vivo* and *in vitro* conditions (Pilet et al., 2012; Marcelino et al., 2015). Our game shows that the aggressiveness of an obligate intracellular pathogenic bacterium is host-triggered and that modifying the host's defense system can lead to an attenuated strain due to an evolutionary process.

By using game theory tools, we provide theoretical basis to the process of generating attenuated strains of obligate intracellular bacterial pathogens, which is already used to develop control strategies such as attenuated vaccines. Moreover, this framework can be used to understand why bacteria can pass from pathogenic to commensal when confronted with an immunocompromised host (Jansen et al., 2015).

## Author contributions

DFM, DT conceived the paper, designed the model, analyzed the results and wrote the paper.

### Conflict of interest statement

The authors declare that the research was conducted in the absence of any commercial or financial relationships that could be construed as a potential conflict of interest.

## Footnotes

^1^Note that the attenuated equilibrium is stabilized for all strains since *c* is an increasing function of the number of times that the replication-without-evasion strategy (strategy B2) has been chosen in the past so in the long run $r^{-}\;>\;\frac{L-c\left(n\right)}{L}$ and the replication-without-evasion strategy (strategy B2) becomes strictly dominant.
